# Ascending and hemi arch replacement during bilateral orthotopic lung transplant: a case report

**DOI:** 10.1093/jscr/rjag965

**Published:** 2026-10-23

**Authors:** Johan van Nispen, Swami Perinkulam, Eric Pruitt, Tomas Martin, Mohammad Aladaileh, Mindaugas Rackauskas

**Affiliations:** University of Florida, Department of Surgery, Division of Cardiac Surgery, PO Box 100286, Gainesville, FL 32610, United States of America; University of Florida, Department of Medicine, Division of Pulmonary and Critical Care Medicine, PO Box 100225, Gainesville, FL 32610-0225, United States of America; University of Florida, Department of Surgery, Division of Cardiac Surgery, PO Box 100286, Gainesville, FL 32610, United States of America; University of Florida, Department of Surgery, Division of Cardiac Surgery, PO Box 100286, Gainesville, FL 32610, United States of America; University of Florida, Department of Surgery, Division of Thoracic Surgery, PO Box 100286, Gainesville, FL 32610, United States of America; University of Florida, Department of Surgery, Division of Thoracic Surgery, PO Box 100286, Gainesville, FL 32610, United States of America

**Keywords:** lung transplant, ascending aortic aneurysm, combined cardiac and thoracic surgery, lung failure

## Abstract

Lung transplant candidates increasingly have concomitant cardiac disease. Prior studies have demonstrated the safety of lung transplant with concurrent cardiac surgery in small case series. Here, we describe a case of ascending aorta replacement at the same time as bilateral lung transplant utilizing a clamshell incision. The patient recovered well. With this case report, we aim to contribute to the growing literature showing the safety of concomitant heart and aortic surgery during lung transplant.

## Introduction

Lung transplant candidates increasingly have concomitant cardiac disease. Prior studies have demonstrated the safety of lung transplant with concurrent cardiac surgery [1, 2] in small case series. Here, we describe a case of ascending aorta replacement at the same time as bilateral lung transplant utilizing a clamshell incision.

## Case report

The patient is a 62-year-old male with Combined Pulmonary Fibrosis and Emphysema (CPFE) who has a DLCO of 22% of predicted on 5 L of home oxygen. CT chest demonstrated bi-apical pleural and parenchymal scarring and reticular changes of the lungs with honeycombing. Echocardiogram demonstrated normal left ventricular function, dilated right ventricle with normal function, and mildly elevated pulmonary artery pressure and moderate aortic insufficiency and ascending aneurysm measuring 4.5 cm. CTA revealed a 5 × 4.5 cm ascending aortic aneurysm (Fig. 1). Cardiac catheterization demonstrated no significant coronary artery disease. The patient was deemed a suitable candidate for transplantation with concomitant treatment of his aneurysm and AI. Lungs became available and he was taken to the operating room. The virtual and actual crossmatches were negative. There was no risk epitope mismatch.

**Figure 1:**
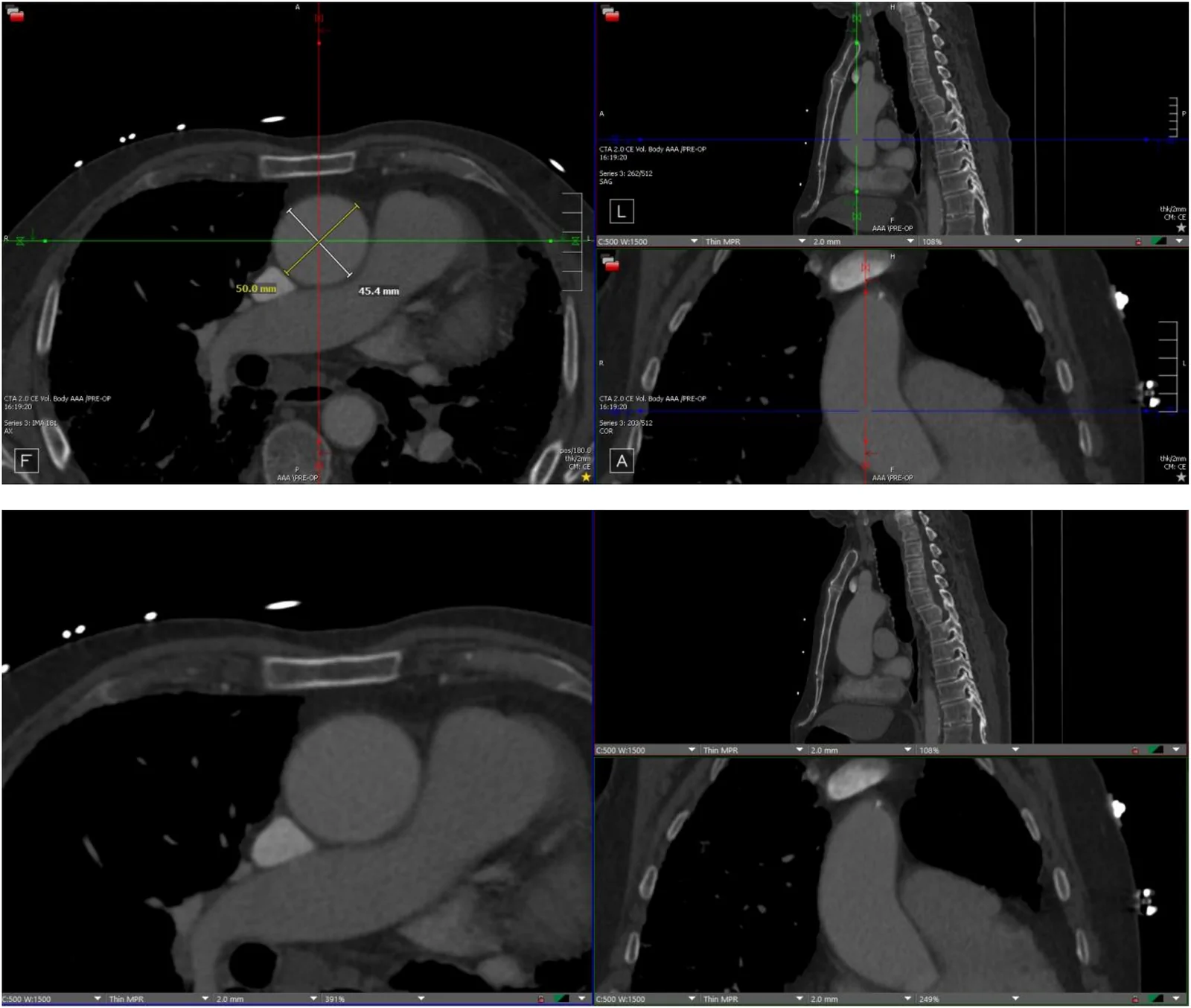
Ascending aortic aneurysm preoperative.

For induction of immunosuppression, he was given basiliximab, 1 g intravenous methylprednisolone, and 1000 mg mycophenolate mofetil. A clamshell thoracosternotomy through the bilateral fourth interspaces was made. The pericardium was opened and the aorta was mobilized to the level of zone 1. Heparin given and epi-aortic ultrasound was utilized to cannulate the ascending aorta across from the base of the innominate artery. A dual stage venous cannula was placed in the right atrial appendage. Cardiopulmonary bypass was initiated, and an extra-pericardial left ventricular vent was placed through the right superior pulmonary vein. The aortic cross clamp was applied and cardioplegia was given. The patient was cooled to 32C°. The ascending aorta was opened and resected to the level of the sinotubular junction. The leaflets were normal appearing and there was annular dilation. A 32 mm graft was anastomosed to the distal ascending aorta at the base of the innominate artery. The proximal aorta was then anastomosed to the graft being sure to maintain the geometry of the aorta. The graft was deaired and the cross clamp was removed. The patient was re warmed. The AI improved to mild. The remainder of the lung transplant commenced. A right pneumonectomy was performed. The hilum and donor lung was prepared, and the right lung was transplanted. Similarly, the left lung was transplanted. The patient was then separated from cardiopulmonary bypass and the cannulas were removed. The patient was temporarily closed to allow for reduction in swelling and improvement of lung compliance. The total cardiopulmonary bypass time was 341 minutes with a cross-clamp time of 35 minutes. The left lung cold ischemia time was 1098 minutes while the right lung cold ischemia time was 992 minutes. He was brought to the intensive care unit on epinephrine drip, vasopressin drip and inhaled nitric oxide. On post operative day (POD) 1, lung volume reduction was performed bilaterally and the chest was closed. Donor serologies for Cytomegalovirus (CMV), Epstein Barr Virus (EBV) and Toxoplasmosis were negative. Recipient was positive for EBV serologies but negative for CMV and toxoplasmosis. He did not have primary graft dysfunction (PGD). Patient had donor derived Hemophilus influenza pneumonia on the immediate post-operative bronchoscopy which was treated with ceftriaxone. On POD #2, the patient was extubated.

He required reintubation for respiratory insufficiency on post operative day #2. After optimal management of volume status and pain, he was successfully extubated again on post operative day #5. He was transitioned to room air on post operative day #7. He was maintained on standard triple immunosuppressive regimen consisting of tacrolimus, mycophenolate and prednisone. For anti-infective prophylaxis he received acyclovir, trimethoprim-sulfamethoxazole, inhaled amphotericin with itraconazole. His post-operative bronchoscopies did not show any evidence of anastomotic airway complications and did not reveal any new infections. He was downgraded from ICU level of care on post operative day #11.

He later developed a right-sided pleural effusion requiring pigtail catheter placement. Pleural fluid was exudative by Light’s criteria and negative for infection. Thoracic imaging demonstrated bibasilar infiltrates. Bronchoscopy with bronchoalveolar lavage was negative for infection but revealed granulation tissue at the left mainstem bronchial anastomosis, which was treated with argon plasma coagulation (Fig. 2).

**Figure 2:**
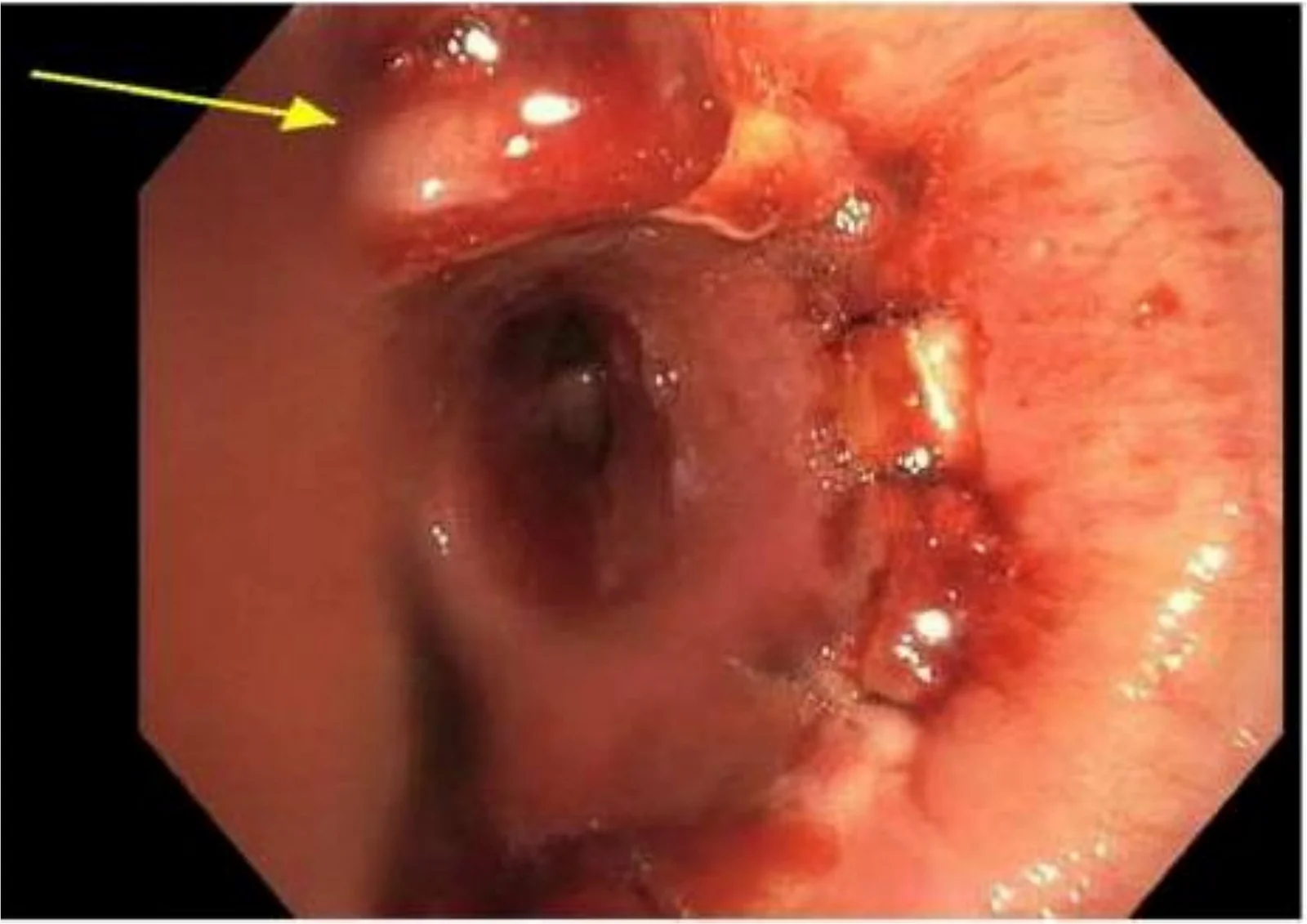
Left mainstem bronchial anastomosis. Arrow indicated area of granulation tissue which was treated with argon plasma coagulation.

Given concern for acute cellular rejection, he received empiric pulse-dose corticosteroids. The pleural effusion reaccumulated after initial catheter removal, leading to hypoxemia requiring high-flow nasal cannula and ICU transfer; a pigtail catheter was reinserted, and diuresis was initiated. Transthoracic echocardiography demonstrated normal cardiac function. His course was further complicated by transient acute kidney injury and hyperkalemia, both managed medically. He required a second ICU admission for hypercapnic respiratory failure necessitating brief endotracheal intubation due to oversedation; however, he was extubated after 2 days.

He underwent intensive rehabilitation and was discharged on postoperative day 50. On follow-up in the clinic, allograft function has progressively improved on pulmonary function testing. Surveillance bronchoscopies have remained negative for infection and rejection, and donor-derived cell-free DNA levels have consistently remained <0.9%. Follow up CT scans without contrast have demonstrated successful treatment of his aneurysm (Fig. 3).

**Figure 3:**
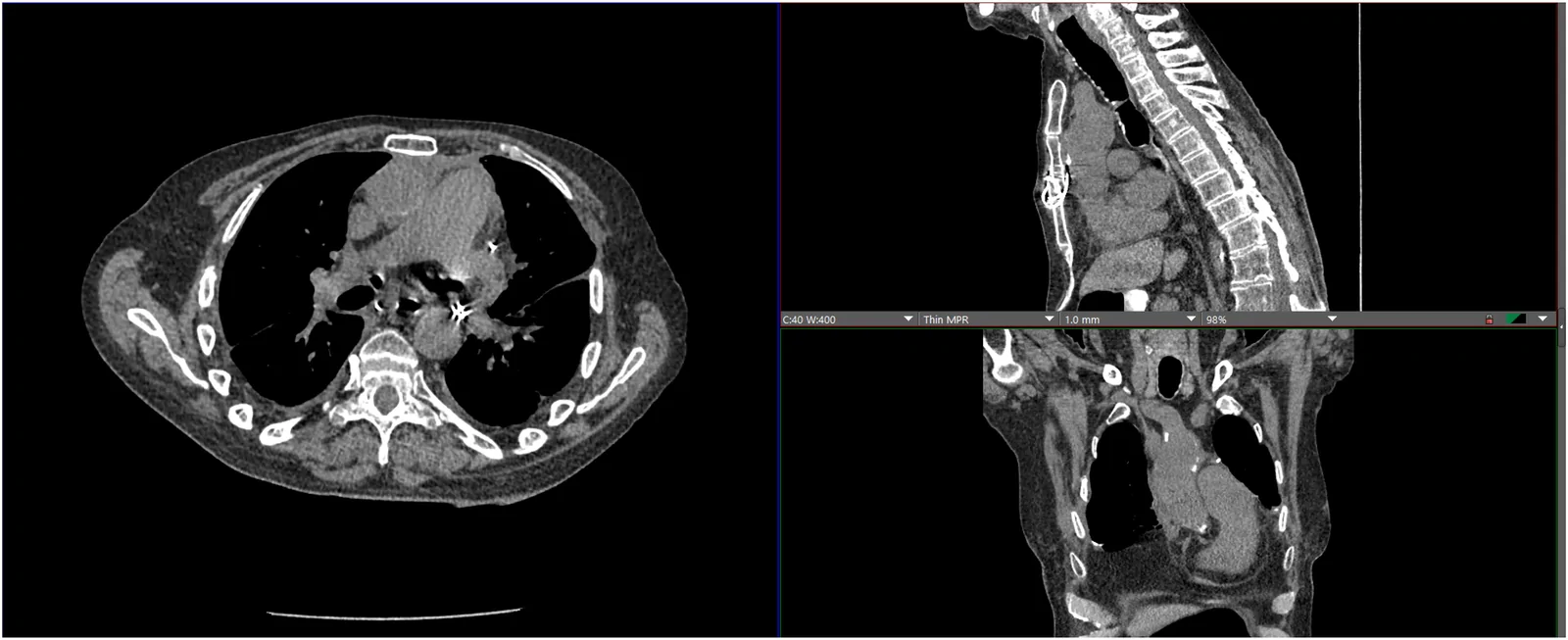
Post operative CT scan of chest demonstrating treated aortic aneurysm.

## Discussion

Concomitant heart surgery with lung transplant is uncommon. Several reports are available regarding the management of coronary artery disease in lung transplant patients including performing bypass surgery during the transplant operation [3]. Data regarding management of aortic disease during lung transplant are lacking. Here we describe an ascending and hemi arch replacement through a clamshell incision with a successful discharge and good outcome, suggesting that concommitent treatment should be further explored.
